# The Role of Nuclear Factor-Kappa B in Fibrinogen-Induced Inflammatory Responses in Cultured Primary Neurons

**DOI:** 10.3390/biom12121741

**Published:** 2022-11-23

**Authors:** Nurul Sulimai, Jason Brown, David Lominadze

**Affiliations:** 1 Department of Surgery, University of South Florida Morsani College of Medicine, Tampa, FL 33612, USA; 2 Department of Molecular Pharmacology and Physiology, University of South Florida Morsani College of Medicine, Tampa, FL 33612, USA

**Keywords:** neuroinflammation, transcription factor, C–C chemokine ligand-2, interleukin-6, intercellular adhesion molecule-1 and cellular prion protein

## Abstract

Traumatic brain injury (TBI) is an inflammatory disease associated with a compromised blood–brain barrier (BBB) and neurodegeneration. One of the consequences of inflammation is an elevated blood level of fibrinogen (Fg), a protein that is mainly produced in the liver. The inflammation-induced changes in the BBB result in Fg extravasation into the brain parenchyma, creating the possibility of its contact with neurons. We have previously shown that interactions of Fg with the neuronal intercellular adhesion molecule-1 and cellular prion protein induced the upregulation of pro-inflammatory cytokines, oxidative damage, increased apoptosis, and cell death. However, the transcription pathway involved in this process was not defined. The association of Fg with the activation of the nuclear factor-κB (NF-κB) and the resultant expression of interleukin-6 (*IL-6*) and C–C chemokine ligand-2 (*CCL2*) were studied in cultured primary mouse brain cortex neurons. Fg-induced gene expression of *CCL2* and *IL-6* and the expression of NF-κB protein were increased in response to a specific interaction of Fg with neurons. These data suggest that TBI-induced neurodegeneration can involve the direct interaction of extravasated Fg with neurons, resulting in the overexpression of pro-inflammatory cytokines through the activation of transcription factor NF-κB. This may be a mechanism involved in vascular cognitive impairment during neuroinflammatory diseases.

## 1. Introduction

Traumatic brain injury (TBI) is an inflammatory neurodegenerative disease that is a leading cause of morbidity and mortality in the United States. Unintentional falls and motor vehicle accidents are the most common cause of injury contributing to a TBI-related hospitalization [1]. The impact of a single fall on these patient’s life could be detrimental, life-altering, or even fatal. Immediate physical damage and inflammation during TBI could ignite a secondary injury and result in neurodegeneration with cognitive deterioration that could show up weeks, months, or even years later. In some cases, TBI patients can have long-term sequelae including cognitive dysfunction, pain, sleep disorders, and physical disability, collectively known as post-concussion syndrome [2]. Although it is known that the blood–brain barrier (BBB) disruption is an early event during TBI, it may also persist for many years after the initial injury [3].

Fibrinogen (Fg) is an acute-phase reactant protein that is increased during the inflammatory condition [4] that accompanies TBI [5]. We have previously found that during an elevated level of Fg, called hyperfibrinogenemia (HFg), there is enhanced cerebrovascular permeability via caveolar protein-mediated transcytosis and the enhanced formation of Fg-amyloid β and Fg-cellular prion protein (PrP^C^) complexes [6]. Extravascular deposits of Fg have been found in a post-mortem brain sample of a TBI patient who survived 18 years after a fall [3]. During chronic inflammatory diseases, such as Alzheimer’s disease (AD) or TBI, the finding of Fg, a protein that is mainly produced in the liver [7], in the extravascular space of the brain parenchyma implies that Fg extravasated from the blood vessels. We have shown that at elevated levels, Fg crosses the vascular wall via, mainly, caveolar transcytosis [8,9,10]. The extravasated Fg that was found in TBI patients [3] certainly can come in contact with neurons and trigger a subsequent signaling responses. We have previously shown that Fg caused the upregulation of pro-inflammatory cytokines in astrocytes [11,12] and neurons [13]. Furthermore, Fg caused an increased generation of reactive oxygen species (ROS) and nitric oxide (NO) in astrocytes [11], in addition to increased ROS, NO, and mitochondrial superoxide in neurons [13]. These pro-inflammatory effects were in part due to the strong association of Fg with its receptors intercellular adhesion molecule-1 (ICAM-1) and PrP^C^ in astrocytes [11] and neurons [13], which ultimately resulted in increased cell death in astrocytes [11] and neurons [13]. These effects could be a mechanism of neurodegeneration and the resultant memory reduction seen during TBI [6,12]. However, the possible transcription pathway that can be activated in neurons because of an interaction with Fg has never been described. Therefore, in the present work, we investigated whether there is a possible role of nuclear factor-κB (NF-κB) in Fg/neuron interactions. To eliminate any confounding effects from other cells, such as endothelial cells, astrocytes, and other glial cells, we conducted the experiments in vitro, testing the effects of intact Fg and mouse brain neurons.

NF-κB is a ubiquitous transcription factor that is one of the most important modulators of stress and inflammatory gene expression in the nervous system [14]. The activation of the NF-κB is associated with many neurodegenerative diseases including TBI [14,15,16]. A prolonged activation of NF-κB has also been found up to 1 year after a head injury [15]. However, the specific source of the persistent post-traumatic activation of NF-κB in this study was not clear. The possible roles of transcription factors and the particular interconnection of Fg and NF-κB signaling in neurodegenerative pathology are not known. The objective of the present study was to investigate the involvement of NF-κB in the pro-inflammatory effects of Fg initiated in the microvasculature that result in the short-term memory reduction that we have shown earlier [5,6,12,17].

## 2. Materials and Methods

### 2.1. Cells, Antibodies, Reagents, and Materials

Primary mouse brain cortical neurons from C57BL/6 embryonic-day-17 mice (cat. #A15586) were purchased cryopreserved from Thermo Fisher Scientific (Waltham, MA, USA). Nunc™ 12-well (cat. #150628) and 24-well plates (cat. #142475), German #1 glass coverslips (cat. #50-121-5159), phosphate-buffered saline (PBS) (composition: 1.05 mM KH2PO4, 155.17 mM NaCl, and 2.97 mM Na_2_HPO_4_. 7H_2_O, without Ca^2+^ and Mg^2+^), ACROS Organics™ Triton™ X-100 (cat. #AC422355000), normal goat serum (cat. #NC9660079), PowerUp™ SYBR™ Green Master Mix (cat. #A25776), Pierce^®^ Radioimmunoprecipitation (RIPA) lysis and extraction buffer (cat. #89900), Laemmli sodium dodecyl-sulfate (SDS) buffer (6X; cat. #J61337), Electron Microscopy Sciences 32% Paraformaldehyde (formaldehyde) aqueous solution (cat. #50-980-494), and Molecular Probes™ ProLong™ Diamond Antifade Mountant with 4′,6-diamidino-2-phenylindole (DAPI) (cat. #P36971) were also from Thermo Fisher Scientific. Primary neuron basal medium (PNBM) (cat. #CC-3256) with a specific additive SingleQuots^TM^ (cat. #CC-4462) was purchased from Lonza (Basel, Switzerland). Poly-d-Lysine hydrobromide (cat. #P0899) and Laminin from Engelbreth-Holm-Swarm murine sarcoma basement membrane (cat. #L2020-1MG), Hirudin from leeches (cat. #H7016-10UN), bovine serum albumin (BSA) (cat. #A7906-10G), the primary antibody against NF-κB p65 (cat. #06-418) that was used in immunofluorescence, and the primary antibody against β-actin (cat. #A1978-200 μL) were purchased from Millipore-Sigma (Burlington, MA, USA). The primary antibody against NF-κB p65 (cat. #06-418) that was used in Western blot was from the Proteintech Group (Rosemont, IL, USA). The inhibitor of NF-κB p65 activity, caffeic acid phenethyl ester (CAPE) (cat. #2743/10), was purchased from R&D systems (Minneapolis, MN, USA). The rat purified function-blocking antibody (clone: YN1/1.7.4) against mouse ICAM-1 (cat. #116133) was purchased from BioLegend (San Diego, CA, USA), and the prion protein-blocking peptide (cat. #GTX89339-PEP) was purchased from GeneTex (Irvine, CA, USA). Recombinant murine tumor necrosis factor alpha (TNFα) (cat. #315-01A-20UG) and interferon gamma (IFNγ) (cat. # 315-05-20UG) were purchased from Peprotech (Rocky Hill, NJ, USA).

The polyclonal rabbit antibody against human Fg (cross-reacts with mouse) (cat. #A008002-2) was purchased from Dako Cytomation (Carpentaria, CA, USA). TRIzol™ reagent (cat. #15596026), ProLong™ Diamond antifade mountant with 4′,6-diamidino-2-phenylindole (cat. # P36962), the primary antibody against neuronal marker microtubule associated protein 2 (MAP2) (cat. #PA1-10005), goat anti-rabbit IgG (H+L) cross-adsorbed secondary antibody, horseradish peroxidase-conjugated (HRP) (cat. #A16104), goat anti-mouse IgG (H+L) cross-adsorbed secondary antibody, HRP (cat. #A16072), goat anti-chicken IgY (H+L) secondary antibody, Alexa Fluor™ 488 (cat. #A-11039), and goat anti-rabbit IgG (H+L) cross-adsorbed secondary antibody, Alexa Fluor™ 647 (cat. #A-21244) were from Invitrogen (Carlsbad, CA, USA). iScript™ cDNA synthesis kit (cat. #1708891), 10% Mini-PROTEAN TGX Stain-Free^TM^ Gels (cat. #4568033), Immun-Blot^®^ polyvinylidene difluoride membranes (cat. #162-0175), and the Quick Start™ Bradford protein assay kit (cat. #5000201) were from BioRad (Hercules, CA, USA). The TransAM^®^ NF-κB p65 activation assay kit (cat. #40096) was purchased from Active Motif (Carlsbad, CA, USA). 

### 2.2. Experimental Procedures with Cell Cultures

Primary mouse brain cortex neurons were plated at 7 × 10^5^ cells/mL, grown in PNBM containing SingleQuots^TM^, and used on day 7 or 10. The neurons were grown on 24-well plates for gene analysis and cell immunocytochemistry and on 12-well plates for protein level detection. The cell culture plates with integrated #1 German glass coverslips were coated with poly-d-Lysine (30 μg/mL) and laminin (200 μg/mL) for 1 h, at room temperature, before being rinsed twice with PBS prior to cell seeding for optimal neuronal attachment and growth. 

The neurons were grown and treated as we reported previously [13]. Prior to treatment with Fg, the neurons were pre-treated for 90 min with a potent and specific inhibitor of nuclear transcription factor NF-κB activation, caffeic acid phenethyl ester (CAPE) [18]. A function-blocking antibody against ICAM-1 or a function-blocking peptide against PrP^C^ (7 μg/mL, for both) was added to the cells 30 min prior to the treatment with Fg. The neurons were treated with Fg for 1 h. In the control group, an equal volume of PBS was added in place of Fg. As a positive control group, cells stimulated with 200 ng/mL of TNFα along with 200 ng/mL of a murine IFNγ were used. Each experimental group contained hirudin (0.5 U/mL) in order to block the possible thrombin-induced conversion of Fg into fibrin. The cells were kept in an incubator at 37 °C with 5% CO_2_. 

### 2.3. Quantitative Real-Time PCR (qRT-PCR) 

Total RNA was extracted from mouse astrocytes using the TRIzol reagent (Invitrogen), and reverse transcription was conducted using an iScript cDNA synthesis kit from Bio-Rad (Hercules, CA, USA), following the manufacturer’s instructions. The qRT-PCR analysis was carried out using PowerUp™ SYBR™ Green Master Mix (Applied Biosystems, Austin, TX, USA). The PCR cycle parameters were 50 °C for 2 min, followed by 95 °C for 10 min, then 40 cycles at 95 °C for 15 s; the annealing temperature was 56 °C for 1 min. The gene expression levels were determined by QuantStudio 3 from Life Technologies (Carlsbad, CA, USA). The mRNA expression of the target genes was analyzed and normalized to that of 18S, which was used as the housekeeping gene. Data analysis of fold changes in gene expression was performed using the ΔΔCt method and is presented as 2- (average ΔΔCt). The primers used were: *CCL2*—Fwd 5′-GTTGGCTCAGCCAGATGCA-3′, Rev 5′-AGCCTACTCATTGGGATCATCTTG-3′; *IL-6*—Fwd 5′-GACTTCCATCGAGTTGCCTTCT-3′, Rev 5′-TTGGGAGTGGTATCCTCTGTGA-3′; and *18S*—Fwd 5′-CGGCGACGACCCATTCGAAC-3’, Rev 5′-GAATCGAACCCTGATTCCCCGTC-3′ as a housekeeping gene.

### 2.4. NF-κB DNA-Binding Activity

#### 2.4.1. Preparation of Nuclear Proteins

Nuclear extracts were collected from treated neurons following the manufacturer’s protocol (Active motif). The neurons were washed with cold PBS/protease inhibitor (PI) (dilution—1:100), scraped, and centrifuged (300× *g* for 5 min at 4 °C). The pelleted neurons were resuspended in 1 mL of hypotonic buffer (HB). HB was prepared with 20 mM Hepes, 5 mM NaF, 10 μM Na_2_MoO_4_, and 0.1 mM EDTA in distilled water adjusted to a pH of 7.5 with NaOH. HB was sterilized by filtering through a 0.2 μm filter and stored at 4 °C until used. The neurons in HB were allowed to swell on ice for 15 min before adding 50 μL of 10% Nonidet P-40 (0.5% final concentration) and vortexing vigorously for 10 s. At this point, the cell membrane was completely lysed, while the nuclear membrane remained intact. The homogenate was centrifuged for 30 s at 4 °C in a microcentrifuge. The supernatant containing the cytoplasmic fraction was removed. The remaining pellet was resuspended in 50 μL of complete lysis buffer and agitated on ice for 30 min on a shaking platform. After being centrifuged for 10 min at 14,000× *g* at 4 °C, the supernatant containing the nuclear extract was collected and stored at −80 °C. 

#### 2.4.2. NF-κB p65 Transcription Factor Detection and Quantification 

To test for DNA binding, the TransAM p65 activation assay kit (Active Motif) was used. The kit contains a 96-well plate coated with DNA oligonucleotides containing the NF-κB consensus site (5′-GGGACTTTCC-3′) that has been immobilized. We added 5 μg of nuclear extract to each well, and the assay was performed as recommended by the manufacturer (Active Motif). The active form of NF-κB in the nuclear extract that was added specifically bound to the oligonucleotide. After the washing steps, we added a primary antibody used to detect NF-κB p65 that recognizes an epitope on p65 that is accessible only when NF-κB is activated and bound to its target DNA. A secondary HRP-conjugated antibody was used that provided a sensitive colorimetric readout quantified by spectrophotometry. The optical density was measured at 450 nm with an absorbance plate reader and is reported as fold increase with respect to the control group.

### 2.5. Western Blot Analysis

The neurons were rinsed with cold PBS/PI (1:100 dilution) twice before lysis with the lysing buffer RIPA/PI (1:100). The content of proteins was assessed with a Bradford assay according to the manufacturer’s protocol. The lysate of neurons was mixed with an equal volume of 6X Laemmli SDS sample buffer and boiled at 105 °C for 10 min. Equal amounts of protein samples of neurons were loaded onto 10% SDS-polyacrylamide gels and electrophoresed under reducing conditions. After electrophoresis, the sample proteins were transferred onto polyvinylidene difluoride membranes. The membranes were blocked with 5% BSA in TBS-T and then were incubated with antibodies against NF-κB p65 (1:1000, host species rabbit) and β-actin (1:5000, host species mouse) for 2 h at room temperature. After probing with goat anti-rabbit IgG (H+L) cross-adsorbed secondary antibody, HRP (1:10,000) and goat anti-mouse IgG (H+L) cross-adsorbed secondary antibody, HRP (1:20,000) for 1 h at room temperature, the blots were developed using a Bio-Rad Molecular Imager (ChemiDoc XRS+, Hercules, CA, USA). Image analysis was performed using Image Lab TM Version 6.0.1 build 34, Standard edition, 2017, Bio-Rad Laboratories, Inc. The results are expressed as a ratio of the integrated optical density (IOD) of the target protein band to the IOD of the β-actin band in the same lane.

### 2.6. Immunofluorescence Staining

The neurons were treated with 1 mg/mL of Fg or with PBS (control) for 60 min. After the incubation, the cells were washed with PBS and fixed with 4% paraformaldehyde in PBS for 15 min. The fixed neurons were treated with a permeating solution containing 0.1% Triton™ X-100 for 10 min before blocking with 3% bovine serum albumin in PBS for 1 h. The neurons were incubated with primary antibodies against MAP2, used at 1:500 dilution, and NFκB p65, used at 1:150 dilution, and kept in the dark at 4 °C overnight. The cells were incubated for 2 h at a 1:200 dilution of an appropriate secondary antibody that was conjugated with Alexa Fluor, Goat anti Rabbit 594 and Goat anti chicken 488. The cells were washed with PBS between the incubations. A mounting medium with DAPI was used to label the cell nuclei. 

### 2.7. Image Analysis

The immunofluorescent-stained neurons were observed using an Olympus FV1000 (Olympus Corporation, Tokyo, Japan) laser-scanning confocal microscope, and the obtained images were deconvoluted using the 2D deconvolution algorithm of the CellSens Dimension 1.11 software (Olympus). NF-κB p65 nuclear translocation was revealed by its co-localization with DAPI.

### 2.8. Statistical Analysis

The data were analyzed using Graph Pad Prism software (San Diego, CA, USA). All data are expressed as mean ± SE. The experimental groups were compared using one-way ANOVA with a pairwise comparison using Tukey’s multiple comparison. Differences were considered significant when *p* < 0.05.

## 3. Results

### 3.1. Fg-Induced Expression of NF-κB p65 in Neurons

**Figure 1:**
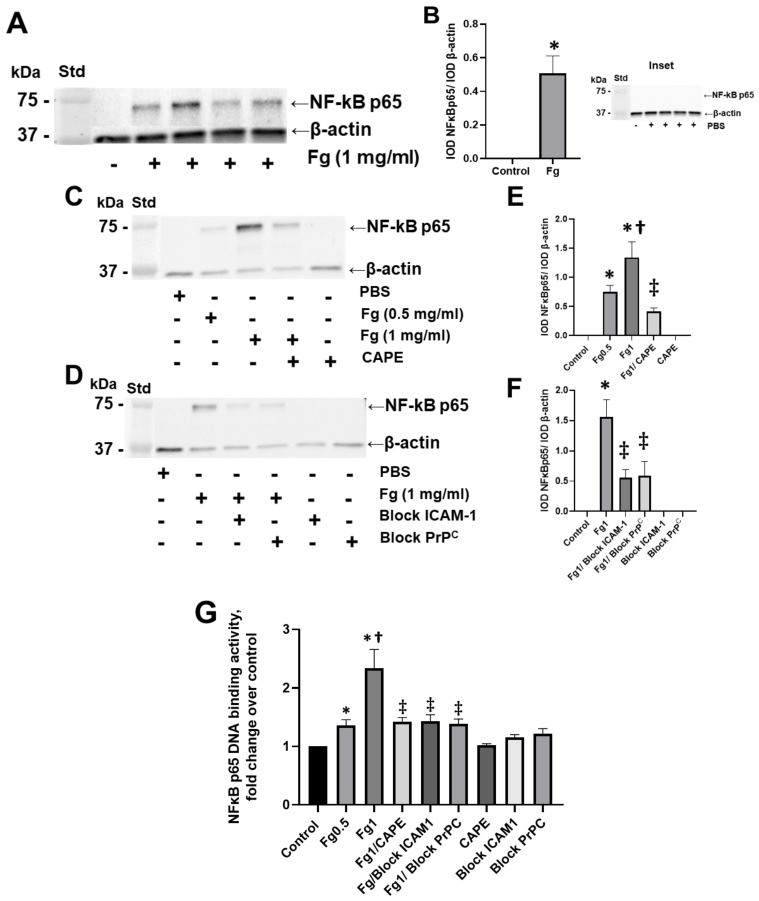
Fibrinogen (Fg)-induced expression of nuclear factor-κB (NF-κB) p65 protein in neurons. Primary mouse brain cortex neurons were treated with 1 mg/mL of Fg or phosphate-buffered saline (PBS) for 1 h. (**A**) The level of the NF-κB p65 protein in neurons was assessed by western blot analysis in Fg-treated and PBS-treated (inset) neurons. (**B**) Summary of the ratios of the integrated optical density (IOD) of NF-κB p65 bands to IOD of β-actin (used as a loading control) bands in their respective lanes. (**C**) Expression of NF-κB p65 proteins in neurons treated with PBS, 0.5 mg/mL of Fg, or 1 mg/mL of Fg in the presence or absence of caffeic acid phenethyl ester (CAPE). (**D**) Expression of NF-κB p65 protein in neurons treated with PBS or 1 mg/mL of Fg in the presence or absence of a function-blocking antibody against ICAM-1 or a function-blocking peptide against PrP^C^. (**E**,**F**) Summary of the ratios of IOD of NF-κB p65 bands to IODs of β-actin bands in their respective lanes. (**G**) Expression of the NF-κB p65 protein in Fg-treated neurons analyzed by the TransAM p65 activation assay (an ELISA based method). The optical density was measured at 450 nm. Molecular weight standard (Std). *p* < 0.05 for all; * vs. control; † vs. Fg0.5; ‡ vs. Fg1; n = 3.

Fg dose-dependently increased the expression of the NF-κB p65 protein in neurons, as shown by Western blot analysis (Figure 1A–C,E). NF-κB p65 protein expression was practically undetectable in neurons treated with vehicle (PBS, control) (Figure 1, inset). Pre-treatment of the cells with CAPE reduced Fg-induced neuronal NF-κB p65 protein expression (Figure 1C,E). Similarly, the use of a function-blocking antibody against ICAM-1 and a function-blocking peptide against PrP^C^ ameliorated Fg-induced NF-κB p65 expression (Figure 1D,F).

### 3.2. Effect of Fg on NF-κB p65 DNA-Binding Activity in Neurons

The results of an ELISA-based assay showed that NF-κB p65 DNA-binding activity was dose-dependently increased in Fg-treated neurons compared to the control group (Figure 1G). The use of CAPE (an inhibitor of NF-κB activation), a function-blocking antibody against ICAM-1, and a function-blocking peptide against PrP^C^ resulted in a reduction of Fg-induced p65 DNA-binding activity (Figure 1G).

### 3.3. Fg-Induced Translocation of NF-κB p65 into the Nucleus

**Figure 2:**
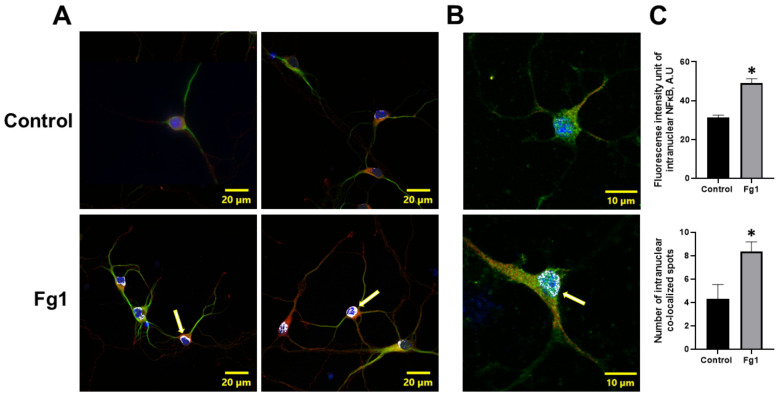
Fibrinogen (Fg)-induced expression of nuclear factor-κB (NF-κB) p65 and its translocation to the nuclei of neurons. Primary mouse brain cortex neurons were treated with 1 mg/mL of Fg (Fg1) or with phosphate-buffered saline (control) for 1 h. (**A**) Representative images show the translocation of NF-κB p65 (red) to the nuclei of Fg-treated neurons defined with a neuronal marker for microtubule-associated protein 2 (MAP2, green). Colocalization of NF-κB with the cell nuclei marked with 4′,6-diamidino-2-phenylindole (DAPI, blue) shown as white (yellow arrows). (**B**) Representative images of the same process at a higher magnification. (**C**) Summary of the fluorescence intensity changes of intranuclear NF-κB staining (upper graph) and the number of spots of co-localized NF-κB and DAPI (lower graph). * *p* < 0.05 vs. control; n = 3.

The expression of NF-κB p65 in the nuclei of neurons treated with 1 mg/mL of Fg was greater than that in the nuclei of neurons treated with vehicle (control) (Figure 2). At the same time, the colocalization of NF-κB p65 with the nuclear marker DAPI in neurons treated with 1 mg/mL of Fg was greater than that in neurons treated with vehicle (control) (Figure 2). Both the expression of NF-κB p65 and the co-localization of NF-κB p65 with the nuclear marker DAPI were significantly reduced by caffeic acid phenethyl ester (CAPE), a function-blocking antibody against ICAM-1, or a function-blocking peptide against PrP^C^ in neurons treated with Fg (Figure 1 and Figure S1).

### 3.4. Fg-Induced Expressions of CCL2 and IL-6 mRNAs in Neurons 

**Figure 3:**
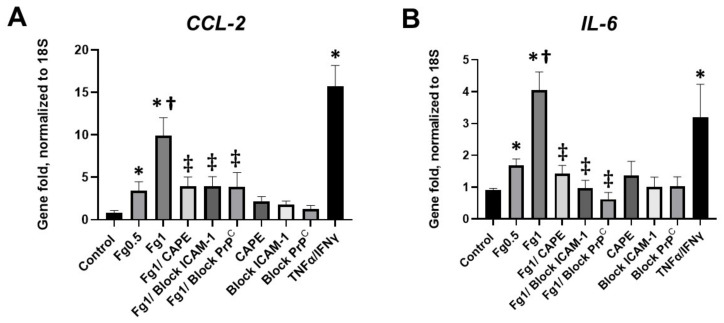
Expression of the mRNA of (**A**) C-C motif chemokine ligand 2 (*CCL2*) and (**B**) interleukin-6 (*IL-6*) in fibrinogen (Fg)-treated neurons, measured by real-time PCR. Primary mouse brain cortex neurons were treated with 0.5 mg/mL of Fg or 1 mg/mL Fg in the presence or absence of caffeic acid phenethyl ester (CAPE), a function-blocking antibody against intercellular adhesion molecule-1 (ICAM-1), or a function blocking peptide against cellular prion protein (PrP^C^) for 1 h. Stimulation of neurons with tumor necrosis factor alpha (TNFα) and interferon gamma (IFNγ) was used as a positive control. *p* < 0.05 for all; * vs. control; † vs. Fg0.5; ‡ vs. Fg1; n = 4.

A real-time PCR analysis demonstrated that Fg induced a dose-dependent increase in the expression of *CCL2* mRNA (Figure 3A). Similarly, Fg induced a dose-dependent increase in the expression of *IL-6* mRNA (Figure 3B). The use of CAPE reduced Fg-induced upregulation of *CCL2* and *IL-6* genes. In parallel, Fg-induced upregulation of *CCL2* and *IL-6* genes was reduced with a function-blocking antibody against ICAM-1 and a peptide against PrP^C^ (Figure 3A,B, respectively). 

## 4. Discussion

For the first time, we present evidence that the direct interaction of plasma soluble Fg with neurons in the brain resulted in the activation of the NF-κB transcription factor in these cells. Fg induced an increased expression of NF-κB protein in neurons (Figure 1). The finding that untreated neurons or neurons treated with PBS expressed undetectable NF-κB p65 protein (Figure 1, inset) indicates the specificity of Fg-induced NF-κB activation in neurons in the present study. These results are in agreement with other studies showing that unstimulated cells present a very low level of NF-κB compared to its substantially up-regulated level in Fg-treated endothelial cells [19] or in mononuclear phagocytes [20]. In endothelial cells, 3 mg/mL of Fg caused the activation of NF-κB, and the effect lasted up to 24 h [19], whereas in mononuclear phagocytes, Fg (50 μg/mL) in the presence of Mn^2+^ induced a significant activation of NF-κB [20]. Thus, an interaction of Fg with endothelial cells, mononuclear phagocytes, or neurons positively resulted in the activation of NF-κB in these cells. These results suggest that an interaction of the blood plasma component Fg not only with vascular cells, but also with neurons, resulted in the activation of these cells, which may serve as the mechanism of known deleterious consequences to these neurons.

Although it is known that NF-κB is activated during TBI [14,15,16], the cause of NF-κB activation after injury has never been associated with Fg. During TBI, NF-κB DNA-binding activity in the injured cerebral cortex increased, with peak binding activity at 3 days after injury, and subsided from 10 to 14 days after the injury [16]. Immediately after injury (from 4 to 24 h), NF-κB activity was mainly observed in the neuronal cells of the affected cortex as well as in astrocytes located in the corpus callosum adjacent to the injury [14,15]. A pulse-like pattern of NF-κB activity in microglial cells was also found [14]. In vascular endothelial cells, NF-κB was detected early (1 h after the injury), and its expression persisted up to 1 year [15]. Our findings that Fg extravasates, even after acute inflammation has subsided during TBI [17], may indicate that there is a possibility of a direct interaction of Fg with neurons. As a result, there is a strong possibility that Fg activates NF-κB in neurons during TBI. It is not surprising that, during severe TBI, the activation of NF-κB was detected soon after a head injury and that the extent of that activity was reduced in a relatively short period of about 2 weeks [14,16]. On the other hand, although it is not specifically stated, the presented characteristics of lesions suggest that injury severity was in the mild-to-moderate range, yet the activation of NF-κB persisted for about one year [15], suggesting that chronic inflammation that lasts that long during mild-to-moderate TBI can still have pathological effects on neurons. Others have shown that BBB disruptions may persist for many years after TBI, and an extensive extravascular Fg disposition has been found in TBI patients who survived 18 years after a fall [3]. Therefore, the long-lasting NF-κB activation contributing to the prolonged (chronic) inflammation and neurodegeneration seen during TBI might be connected, in part, to NF-κB activation in neurons by extravasated Fg and/or its product fibrin. This does not exclude effects of Fg on endothelial cells [19] and mononuclear phagocytes [20] during the neuroinflammatory injury.

Interestingly, blocking the function of ICAM-1 and PrP^C^ dampened NF-κB DNA-binding activity and NF-κB protein expression (Figure 1D,F,G). The specificity of these effects was confirmed by the reduction of intranuclear of NF-κB and its translocation to the nuclei of neurons when the functions of both ICAM-1 and PrP^C^ were blocked (Figure S1). 

The nuclear translocation of the NF-κB subunit p65 is a crucial step in NF-κB pathway activation. Thus, we determined the effects of Fg on NF-κB p65 translocation from the cytoplasm to the nucleus in neurons using immunocytochemistry. NF-κB p65 immunolabelling confirmed that there was an enhanced intracellular translocation of NF-κB p65 in Fg-treated neurons (Figure 2).

In this study, we also found that Fg-induced upregulation of the proinflammatory cytokines *CCL-2* and *IL-6* was ameliorated in the presence of a function-blocking antibody against ICAM-1 or a function blocking peptide against PrP^C^ (Figure 3). Collectively, these results suggest that the interaction of Fg with both its receptors ICAM-1 and PrP^C^ on neurons and the resultant upregulation of the pro-inflammatory cytokines *CCL2* and *IL-6* were associated with NF-κB signaling in these cells.

CCL2 is produced by neurons and glial cells in the brain and has been shown to be upregulated as a result of injury and inflammation [21,22]. Upregulation of CCL2 has also been shown in the cerebrospinal fluid (CSF) from patients with neurodegenerative diseases including mild cognitive impairment and Alzheimer’s disease [23]. At the same time, high Fg levels were associated with an increased risk of both AD and vascular dementia [24]. In the current study, we found that Fg caused the upregulation of neuronal CCL2. We have shown that Fg resulted in the overexpression of *CCL2* in astrocytes [11], and others have shown that Fg increased the expression of CCL2 in endothelial cells [19]. Although these effects did not occur in neurons, they still confirm the pro-inflammatory potential of Fg in the brain during neurodegenerative diseases. Extravascularly, Fg, and most likely its product fibrin, have been shown to trigger the recruitment of inflammatory monocytes into the CNS and to induce demyelination [25]. In patients with AD, there was a positive correlation between cognitive impairment and the CCL2 concentration in the CSF [23]. We previously showed that Fg had a significant role in inflammatory responses leading to impairment in cognition [12]. Fg significantly increased cerebrovascular permeability [6], the formation of Fg–PrP^C^ complexes [26], and the generation of ROS [13] and reduced short-term memory during TBI [5,6,12]. The Fg-induced *CCL2* upregulation in neurons that we found in the current study might be the bridge that connects a pro-inflammatory state to cognitive decline, as we [27] and others have found [23]. Furthermore, CCL2 has been shown to induce an increase in BBB permeability by causing alterations in tight junction proteins in endothelial cells [28,29], which can further contribute to neuroinflammatory processes. Thus, Fg-induced activation of NF-κB could play a role in the signaling mechanism that results in the upregulation of the pro-inflammatory cytokines CCL2 and IL-6, causing the neuropathology seen during neuroinflammatory diseases [23] that is associated with elevated levels of Fg [25]. 

NF-κB is a pleiotropic transcription factor that is widely expressed in all cell types in the brain. In basal conditions, NF-κB functions to maintain healthy neuronal conditions, synapse growth, and plasticity-related functions [30]. In our study, we found that blocking NF-κB activation in Fg-induced neuronal pathology via a specific inhibitor for NF-κB, such as CAPE, or blocking ICAM-1 and PrP^C^ could be beneficial in preventing the activation of neurons caused by the neuroinflammation-induced detrimental effects of an elevated blood level of Fg. It is known that CAPE is also an active component of propolis, a resin-like material made by bees that is known to possess anti-inflammatory and antioxidative properties and is widely used as a therapeutic agent [18]. A comprehensive meta-analysis study confirmed a significant reduction in IL-6 and C-reactive protein levels as a result of propolis consumption [31]. Perhaps, propolis can be a useful remedy to alleviate some Fg-induced pathologies associated with NF-κB activation. This is the first report that connects a neuropathology that involves NF-κB signaling in neurons to the Fg protein that is originated from the vasculature and can extravasate only as the result of abnormal vascular permeability. Moreover, we found that at an elevated level of Fg can itself cause an increase in cerebrovascular permeability mainly via caveolar transcytosis [5,6,9,32,33]. A study of the role of Fg-induced NF-κB activation during TBI may provide opportunities for therapeutic interventions or new insights into the mechanisms of action for agents with therapeutic potential in the prevention of neurodegeneration and the resultant memory reduction as a result of neuroinflammatory diseases. 

Many neuroinflammatory diseases such as TBI [5,34], AD [24], and stroke [35] are accompanied by an increased blood level of Fg. We, and others, have shown that TBI is associated with an enhanced deposition of Fg in the brain tissue [3,17]. There are reports of perivascular deposition of Fg during AD [36] and stroke-associated cerebral infarction [37,38]. We have shown that during HFg, which is a marker of inflammation [4], Fg deposition in the extravascular space is increased [6]. Combined, these results and the results of the present study, indicate that Fg can interact with neurons during any neuroinflammatory disease that is accompanied an increased deposition of Fg in the brain interstitium and results in neurodegeneration through the activation of NF-κB pathway.

## 5. Conclusions

This study demonstrated that Fg dose-dependently induced NF-κB activation in neurons. Furthermore, our results showed that the Fg-induced NF-κB activation may be inhibited by blocking known receptors of Fg (ICAM-1 and PrP^C^), indicating that this is a specific effect of Fg. The present study, for the first time, clarified that blocking the neuronal receptors ICAM-1 and PrP^C^ reduced Fg-induced NF-κB activation and the resultant pro-inflammatory cytokine production. Therefore, this report suggests that using NF-κB -specific anti-inflammatory agents or blocking the function of the Fg receptors ICAM-1 and PrP^C^ should be considered as candidates for the treatment of the Fg-induced neuroinflammatory effects that accompany neurodegenerative diseases and in particular TBI, which is associated with chronic inflammation, deposition of Fg in the brain parenchyma, and increased neurodegeneration resulting in memory reduction. These findings identify possible mechanisms that are involved in the observed effects that originate in the vasculature (vascular permeability, Fg extravasation) and result in neuroinflammation (manifested as the expression of *CCL-2* and *IL-6*), leading to the memory reduction previously found during TBI [6] and collectively called vascular cognitive impairment and dementia. 

## Data Availability

Not applicable.

## Supplementary Materials

The following supporting information can be downloaded at: https://www.mdpi.com/article/10.3390/biom12121741/s1. Figure S1: Reduction of fibrinogen (Fg)-induced expression of nuclear factor-κB (NF-κB) p65, through the application of a NF-κB antagonist and the prevention of Fg/neuron interactions, and its translocation to neuronal nuclei. Figures S2–S6: Original images of Western blots shown in Figure 2.
