# Supplementary material for: The Role of Nuclear Factor-Kappa B in Fibrinogen-Induced Inflammatory Responses in Cultured Primary Neurons
Source: Biomolecules. 2022 Nov 23;12(12):1741. doi: 10.3390/biom12121741 (PMC9775651; doi:10.3390/biom12121741)
Supplement: Supplementary file 1 [file biomolecules-12-01741-s001.zip › biomolecules-2022497-supplementary.pdf]

Supplementary Materials

1. Results and Discussion

1.1. Fg-induced translocation of NF-κB p65 into nucleus

We found that the expression of NFkB p65 in the nuclei of neurons treated with 1mg/ml of Fg was greater than that in the nuclei of neurons treated with vehicle (control). The colocalization of NFkB p65 with the nuclear marker 4′,6-diamidino-2-phenylindole (DAPI) in neurons treated with 1mg/ml of Fg was also greater than that in neurons treated with vehicle (control). Both the expression of NFkB p65 and the co-localization NFkB p65 and nuclear marker DAPI were significantly reduced by caffeic acid phenethyl ester (CAPE), a function-blocking antibody against ICAM-1, or a function-blocking peptide against PrP^C in neurons treated with Fg.

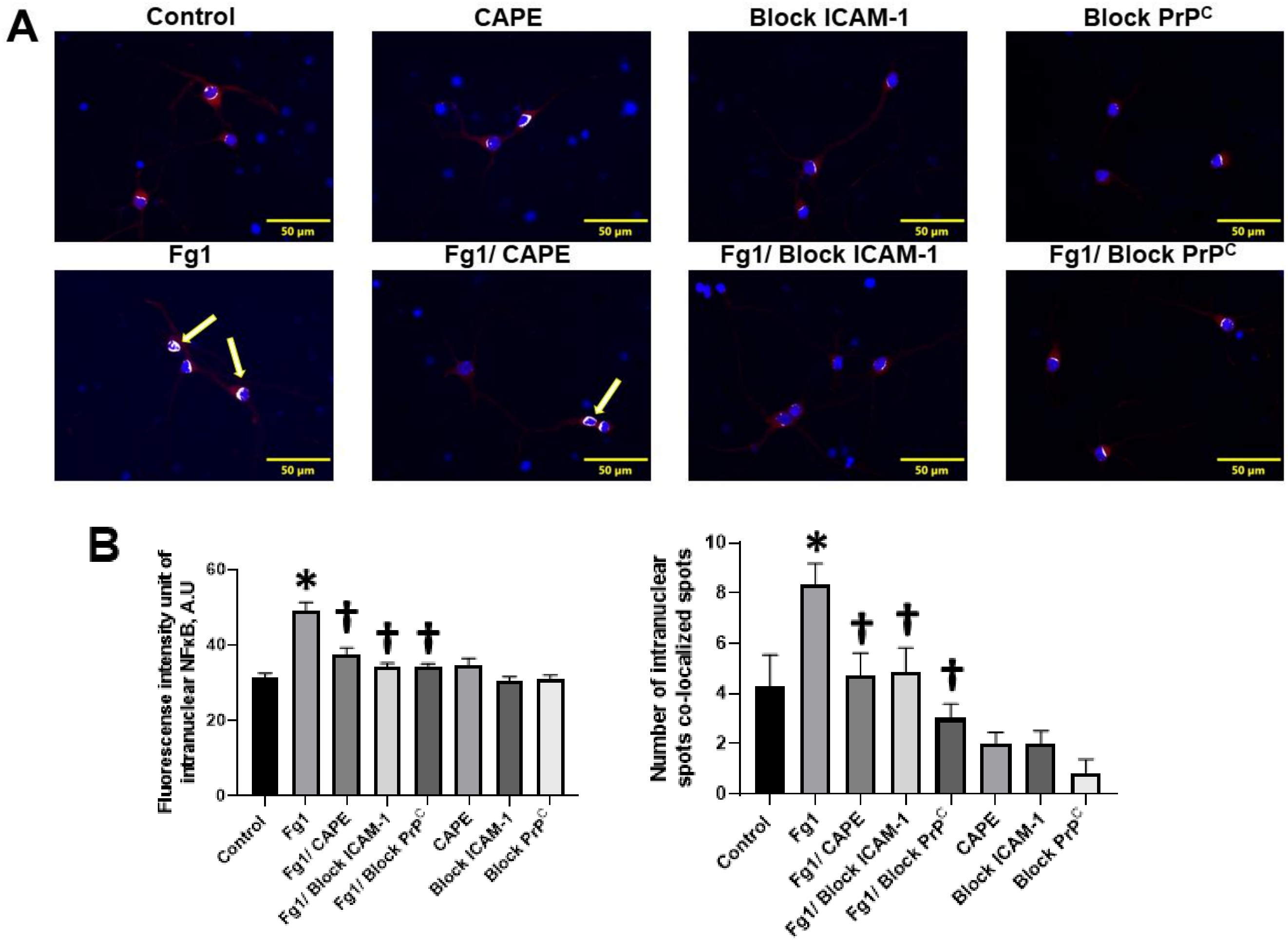

Figure S1. The reduction of fibrinogen (Fg)-induced expression of nuclear factor-κB (NF-κB) p65, through the application of an NF-κB antagonist and the prevention of Fg/neurons interactions, and its translocation to neuronal nuclei. Primary mouse brain cortex neurons were treated with phosphate buffered saline (PBS) or with 1 mg/ml of Fg in the presence or absence of caffeic acid phenethyl ester (CAPE), a function-blocking antibody against intercellular adhesion protein-1 (ICAM-1), or a function-

blocking peptide against cellular prion protein (PrP^C). **(A)** Representative images of the translocation of NF-kB p65 (red) to the nuclei of Fg-treated neurons, defined with a neuronal marker of microtubule-associated protein 2 (MAP2, green). Nuclei of cells are marked with 4′,6-diamidino-2-phenylindole (DAPI, blue). Co-localization of NF-kB p65 and DAPI is shown as a white spot (yellow arrows). **(B)** Summaries of fluorescence intensity changes of intranuclear NF-κB staining (left graph) and the number of co-localized NF-κB and DAPI in the nucleus of a neuron (right graph). $P < 0.05$ for all; ∗ vs. control; † vs. Fg1; n = 3.

2. Original images for Western blot

The full image of western blot with marked NFkB p65 and β-actin.

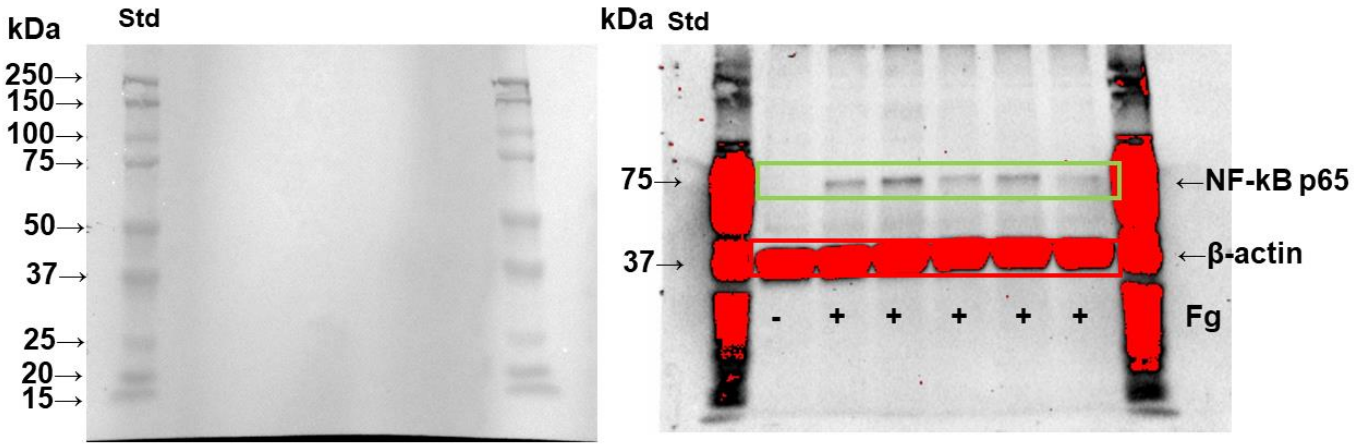

Figure S2. Original western blot image of Figure 2A for NFkB p65.

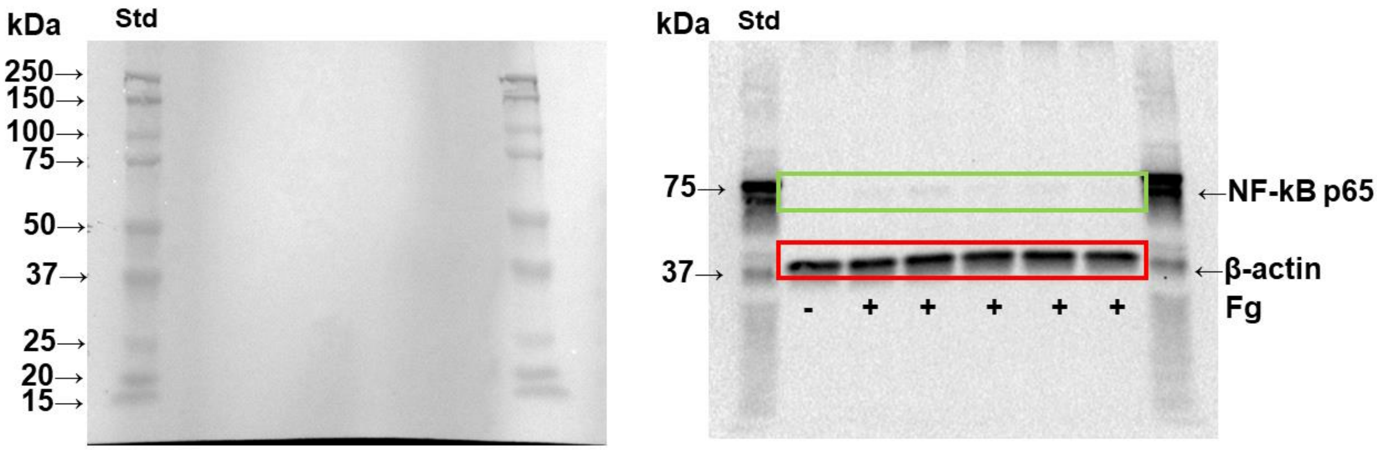

Figure S3. Original western blot image of Figure 2A (longer exposure time for better β-actin detection).

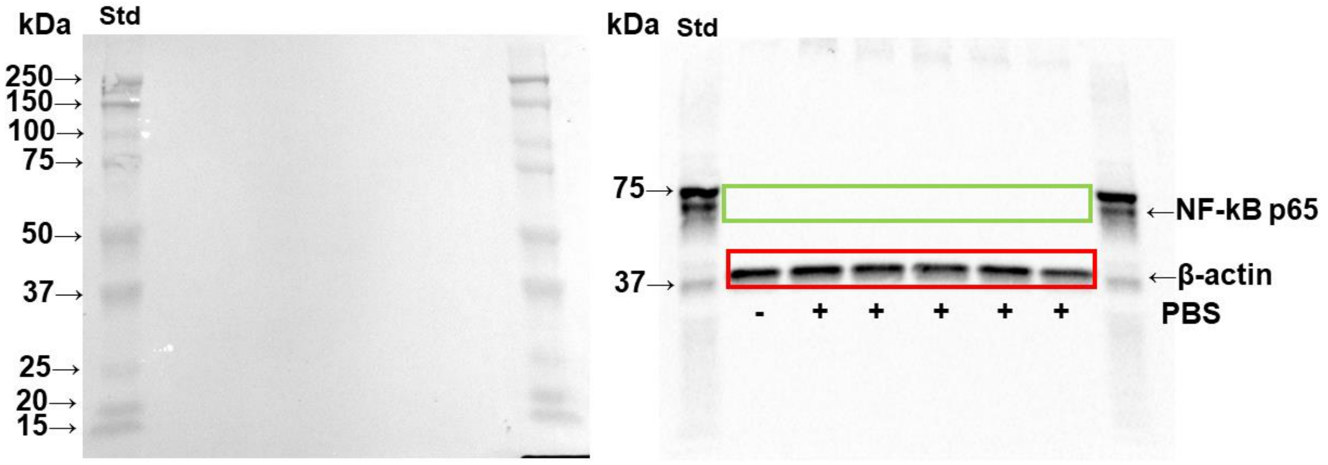

Figure S4. Original Western blot image of Figure 2 inset.

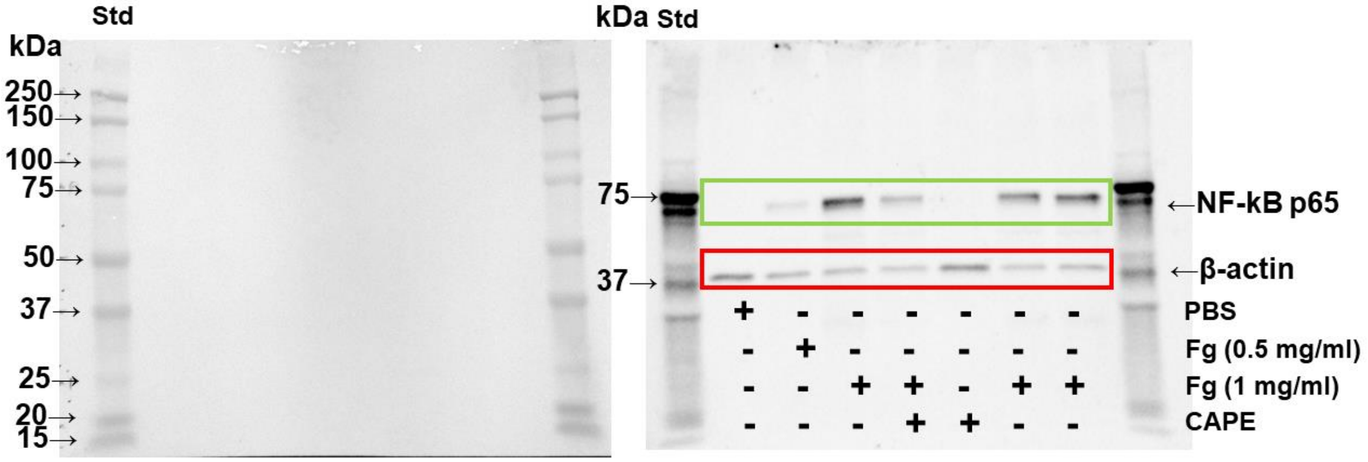

Figure S5. Original Western blot image of Figure 2C.

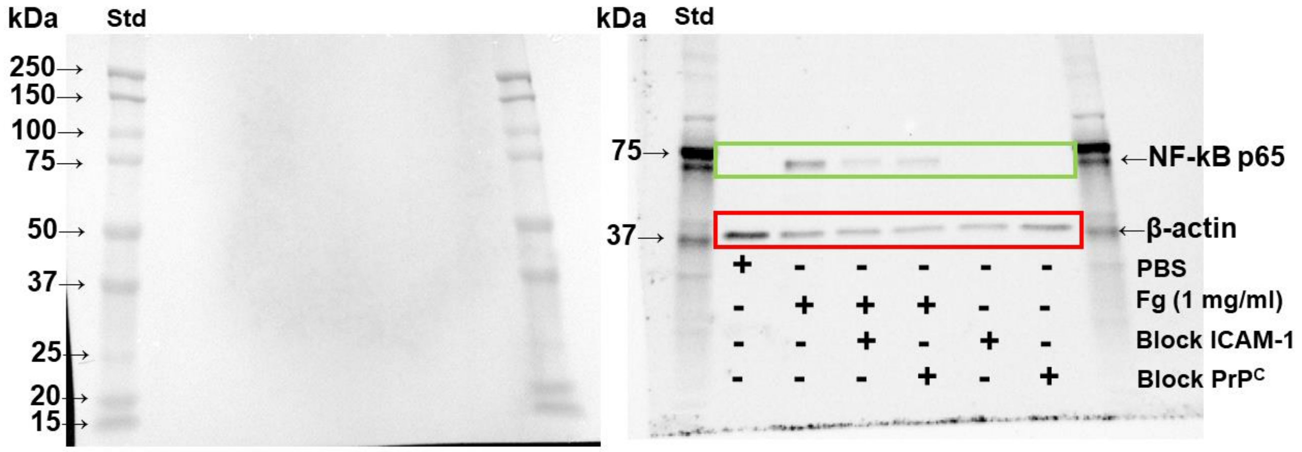

Figure S6. Original Western blot image of Figure 2D.
